# The impact of common redox mediators on cellular health: a comprehensive study†

**DOI:** 10.1039/d5an00017c

**Published:** 2025-03-12

**Authors:** Samuel P. Nortz, Vanshika Gupta, Jeffrey E. Dick

**Affiliations:** a Department of Chemistry, Purdue University West Lafayette IN 47907 USA jdick@purdue.edu; b Elmore Family School of Electrical and Computer Engineering, Purdue University West Lafayette IN 47906 USA

## Abstract

Electrochemistry has become a key technique for studying biomolecular reactions and dynamics of living systems by using electron-transfer reactions to probe the complex interactions between biological redox molecules and their surrounding environments. To enable such measurements, redox mediators such as ferro/ferricyanide, ferrocene methanol, and tris(bipyridine) ruthenium(ii) chloride are used. However, the impact of these exogeneous redox mediators on the health of cell cultures remains underexplored. Herein, we present the effects of three common redox mediators on the health of four of the most commonly used cell lines (Panc1, HeLa, U2OS, and MDA-MB-231) in biological studies. Cell health was assessed using three independent parameters: reactive oxygen species quantification by fluorescence flow cytometry, cell migration through scratch assays, and cell growth *via* luminescence assays. We show that as the concentration of mediator exceeds 1 mM, ROS increases in all cell types while cell viability plumets. In contrast, cell migration was only hindered at the highest concentration of each mediator. Our observations highlight the crucial role that optimized mediator concentrations play in ensuring accuracy when studying biological systems by electrochemical methods. As such, these findings provide a critical reference for selecting redox mediator concentrations for bioanalytical studies on live cells.

## Abstract 2

A compehrensive study on the effects of electrochemical mediators on cellular health that highlights detrimental mediator concentrations shown through studies on reactive oxygen species, cell migration, and cell growth and viability.

## Graphical abstract

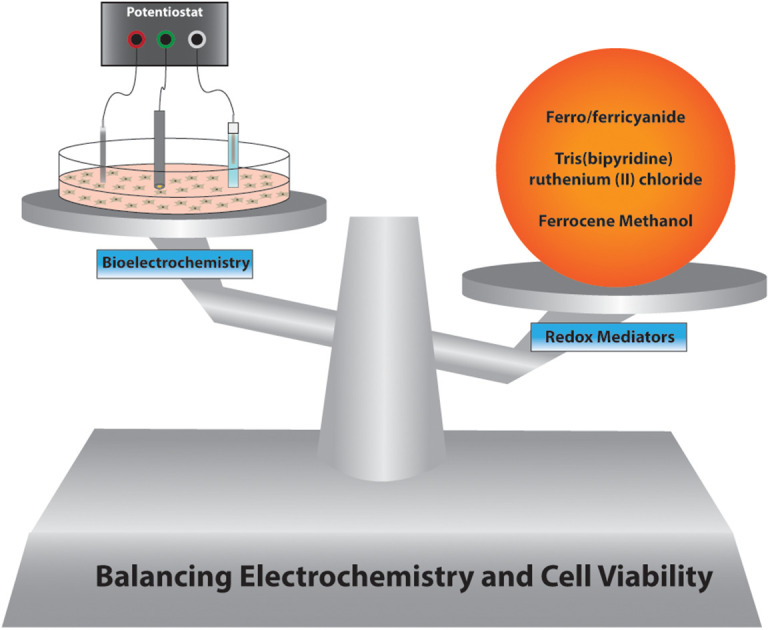

## Introduction

Understanding the dynamic environment of living systems at the molecular level is crucial for advancing the fields of biochemistry,^1^ medicine,^2^ and drug development.^3^ By delving into the molecular mechanisms of compounds such as drug metabolites,^4^ biomarkers,^5^ and proteins,^6^ scientists have helped decode different aspects of diseases such as cancer.^7^ Conventional bioanalytical techniques for the identification and quantification of such compounds include fluorescence,^8,9^ chromatography,^10^ and mass spectrometry.^11^ Although these techniques offer high sensitivity and signal-to-noise, as well as well-established methodologies, they typically provide only a snapshot of analytes in time,^12^ require expensive instrumentation,^13^ and entail slow sample processing.^14^

Electrochemistry has emerged as a pivotal technique with good spatiotemporal resolution^15^ and fast sample processing,^16^ while still maintaining high sensitivity,^17^ specificity,^18^ and signal-to-noise ratio.^18^ Additionally, electrochemical experiments are more cost effective,^19,20^ offer miniaturization capabilities,^21^ and require less sample preparation as measurements can be made directly in complex biological systems.^22^ Common electrochemical techniques for bioanalysis include scanning electrochemical microscopy (SECM),^23–25^ electrochemical biosensors,^26^ electrochemical impedance spectroscopy,^27^ and electrochemiluminescence (ECL).^28,29^ While unique in their applications, each of these techniques requires a working electrode where the desired reaction takes place, a counter electrode held at the opposite potential from the working electrode for charge balance, a reference electrode to poise the potential, and redox mediators or organic small molecules that readily facilitate electron transfer between the solution and electrode interface.^30^ Examples of commonly used electrochemical mediators in electrochemical experiments include ferro/ferricyanide,^31^ ferrocene methanol,^32^ and tris(bipyridine) ruthenium(ii) chloride.^33^ The concentration of exogenous mediators typically used in biological studies falls within micromolar to millimolar range.^34^ In some cases, higher concentrations of the mediator are required to detect low-abundance biological analytes.^35^ Akin to other parameters, the optimization of redox mediator concentration is a key step in electrochemical analysis techniques. However, this optimization is most often performed towards the goal of achieving high signal-to-noise ratios and sensitivity while the impact of these mediators’ concentration on cell viability and function is often overlooked. It is crucial to consider the potential cytotoxic effects of chemical perturbations in bioanalytical studies. Previously, electrochemists have individually reported the impact of electrochemical measurements on cell viability using fluorescent probes to visualize cells^25^ and reactive oxygen species,^36^ but no comprehensive study has reported on the change in cell health as a function of electrochemical mediator use. This study aims to bridge that gap.

Changes in cell health can be quantified by looking at three key parameters: fluctuations in the reactive oxygen species concentration, reduction in migration abilities, and modulations in the growth rate and viability of cells. Reactive oxygen species are a normal byproduct of metabolism in healthy cells but can become harmful when produced in excess. Cell migration is a cell's tendency to move through space to communicate with other cells and maintain homeostasis. Cell growth is a cell's ability to divide and multiply. Common methods to quantify these parameters include fluorescence,^37^ luminescence,^38^ and flow cytometry,^39^ each of which were utilized within this study. Specifically, in fluorescence and luminescence-based assays, a fluorophore or luminophore is used to detect indicators of healthy cellular function, such as cellular membrane integrity,^40^ or metabolic activity.^41^ Using these probes, scientists have uncovered the effects of exogenous compounds, such as drugs, on cell health to establish a benchmark for dosage concentration and exposure time without compromising cell viability.^42^ These experimental design guidelines help to mitigate cytotoxic effects that may negatively impact experimental results. In this study, we measure the effect of redox mediators on various markers of cell health, uncovering cellular responses on the population level in these potentially stressful environments. First, we evaluated the ROS levels by fluorescence flow cytometry to indicate oxidative stress levels. Next, we measured cell migration using a simple scratch assay to characterize changes in cell mobility as a function of mediator concentration. Lastly, we monitored the cell growth during mediator exposure and the cell recovery after mediator introduction through a luminescence-based assay to indicate cell proliferation and viability. Mediator exposure times were chosen to cover the wide range of typical experimentation times previously used in literature. Bioelectrochemical analyses using SECM and ECL are typically completed over a relatively short duration.^43–46^ However, for practical and clinically relevant applications such as pharmacokinetic^47^ and metabolic^48^ studies, longer analysis times are often necessary. Thus, each of the common electroanalytical techniques have the potential to be extended over multiple hour durations, which justifies our investigation up to 8 hours in this study.

Herein, we used four immortalized human cell lines: Panc1 (pancreatic carcinoma), U2OS (osteosarcoma), HeLa (cervical adenocarcinoma), and MDA-MB-231 (breast adenocarcinoma). Each cell line was exposed to varying concentrations of ferrocyanide/ferricyanide (1 : 1 mixture, FiFo), ferrocene methanol (FcMeOH), and tris(bipyridine) ruthenium(ii) chloride (referred throughout as RuBpy) which are the most commonly used electrochemical redox mediators utilized for biological systems.^49^ Through our work we show that the concentration of mediator used has a significant impact on cell health. Specifically, as concentration of the mediator increased, reactive oxygen species increased across all cell lines and redox mediators. Using a cell-viability luminescence assay, increasing the concentration of mediator showed a general trend of hindered cell growth, especially at the highest concentration of each mediator. Cell migration was only hindered at the highest concentration of mediators. This work shows that the concentration of mediator exceeds 1 mM, ROS increases in all cell types while cell viability significantly decreases. The results of this study are unprecedented as they provide a threshold for concentrations of redox mediators that influence cell health in biological analyses.

## Materials and methods

### Materials and reagents

Potassium ferrocyanide (99%) and potassium ferricyanide (99%) were purchased from Thermo Fisher Scientific. Ferrocenemethanol (FcMeOH, 97%) and tris(bipyridine) ruthenium(ii) chloride hexahydrate ([Ru(bpy)_3_][Cl_2_]·6H_2_O, 98%) were purchased from Sigma-Aldrich. All chemicals were used without further purification steps. For cell culture, T75 flasks, 6-well plates, and 96-well plates were purchased from Thermo Fisher. Dulbecco's modified Eagle's medium (DMEM), Dulbecco's phosphate-buffered saline (DPBS), TrypLE Express enzyme, and CellROX green reagent were obtained from Thermo Fisher. NanoLuc® luciferase enzyme and RealTime-Glo™ viability substrate were purchased from Promega, Madison, WI.

### Cell culture

All mammalian cell cultures were procured from ATCC (Manassas, VA) and passaged multiple times before being frozen for later use. All cell cultures used in this experiment were seeded from cell samples stored at −80 °C and maintained in DMEM with 10% FBS and 2% penicillin/streptomycin. The cultures were kept in a stable environment at 37 °C and 5% CO_2_. Cell growth was monitored daily and media was changed accordingly, until cultures reached approximately 80% confluence. Cell cultures were lifted using trypsin, centrifuged for 10 minutes at 1000 rpm, resuspended in fresh media, and either split into new culture flasks or seeded in the appropriate plate for experimentation. The seeding cell density was optimized for each cell line and different plate size to achieve the target confluence before the start of each experiment.

### Reaction oxygen species assay

For the detection of reactive oxygen species, cells were grown to 80–90% confluence and incubated in mediator for 6 hours. The cells were stained with CellROX oxidative stress stain at a concentration of 5 μM of stain and incubated for 30 minutes. Cells were rinsed with DPBS and lifted with trypsin. The cells were centrifuged at 2000 rpm for 8 minutes, resuspended in 0.5% bovine serum albumin in PBS, and stored on ice during transport to the flow cytometer. The analysis was done on a BD LSRFortessa Cell Analyzer (BD Biosciences, Franklin Lakes, NJ). The cell density of all samples run on the flow cytometer was at least 5 × 10^5^ cells per mL.

The same gating was used for all flow cytometry analyses (Fig. S1†). First, we used a gating regime to distinguish live cells from dead cells. Events observed outside the boundaries of the gate were considered dead cells or abiotic particulate matter. Of those events within the live cell gate, we gated for single cellular events. Events outside the chosen gate were considered a doublet (clump of two or more cells). Lastly, we gated for cells emitting the ROS-generated fluorescence. No fluorescence was observed in the unstained control samples (Fig. S1c†). The FITC fluorescence channel with a maximum excitation/emission of 494 nm/519 nm was chosen to detect the oxidative stress stain, which has a maximum excitation/emission of 485 nm/520 nm.

### Growth and recovery assay

A GloMax Explorer microplate reader (Promega, Madison, WI) was used to measure bioluminescence given off by cells. The luciferase enzyme and substrate were used as reagents to produce the luminescence. Cells were seeded in a flat-bottom 96-well plate the day before beginning the experiment between a density of 1000–5000 cells per mL, depending on the cell type. The enzyme and substrate stock solutions were diluted 1000× by adding directly to each of the mediator-cell media treatment solutions. To track their growth, the cells were incubated at 37 °C for 8 hours in the presence of the mediator. Luminescence was measured on the microplate reader every 2 hours.

To track recovery of cell growth following exposure to mediator, cells were grown to roughly 50% confluence. The cells were then incubated in mediator in media for 6 hours. 75% of the media was then removed and replaced with fresh, blank media. This process was done twice more so that only a trace amount of the mediator was leftover in the sample. The enzyme and substrate in fresh media (diluted 1000×) were added to the cells in the final addition of blank media. To track their growth over a 24 hour period, luminescence was measured 6 hours, 18 hours, and 24 hours from the time the enzyme and substrate reagents were added.

### Cell migration assay

To track cell mobility, we used an *in vitro* scratch assay adopted from a previously described method.^50^ Cells were imaged under a Nikon Eclipse Ts2 inverted microscope (Nikon Corporation, Melville, NY). Cells were grown to roughly 80% confluence in 6-well plates and a scratch was made down the center of the plate using a pipette tip to remove cells from a region approximately 1 mm wide. After rinsing each plate with 1 mL PBS, mediator in DMEM was added to the cells. The movement of the cells into the empty scratch was tracked and quantified over a period of 72 hours. Every 24 hours, an image of the scratch was taken using an optical microscope and the media was changed with fresh mediator in DMEM. The point to which cells migrated was defined as the “cellular front”, which was quantified using ImageJ as a percent change in area of the scratch (Fig. 2a).

### Statistical analyses

All statistics were carried out using Microsoft Excel 16.89.1. Data are reported as the means the standard deviation of *n* = 6 sample sizes. Using unpaired *t*-tests, all experimental treatments were compared to the control (0 mM) treatment with probabilities *P* < 0.05 considered statistically significant. Grubb's test was used to identify outliers in each sample set. The sample was removed from the set if the *G*-value was greater than the critical value of *G*(*α*, *n*) = 1.887, where *α* = 0.05 and *n* = 6.

## Results and discussion

### Reactive oxygen species

Reactive oxygen species (ROS) play a major role within cells as both detrimental and beneficial molecules depending on their concentration.^51^ ROS include a variety of diverse chemical species including hydrogen peroxide, hydroxyl radicals, and superoxide anions.^52^ These radical species can be produced intracellularly or exogenously.^51^ The majority of intracellular ROS is generated from the electron-transport chain in the mitochondria and NADPH oxidases in the cytosol as a result of normal cellular metabolism.^52^ An antioxidant defense system of enzymes including superoxide dismutase, catalase, and glutathione peroxidase regulate ROS intracellular ROS levels.^53^ There is growing evidence that shows the role of ROS as a double-edged sword. For example, ROS can be upregulated within cells and cause oxidative stress, a detrimental process that can mediate damage to lipids and cell membranes, proteins, and DNA.^54^ Conversely, ROS at low to moderate concentrations plays a key physiological role in cellular response and signaling, as it can induce apoptosis and act as an anti-tumorigenic species.^54^ Some small molecule chemotherapeutics have been shown to enhance intracellular ROS levels by impairing antioxidant defense systems or directly increasing the generation of ROS.^55,56^

**Fig. 1:**
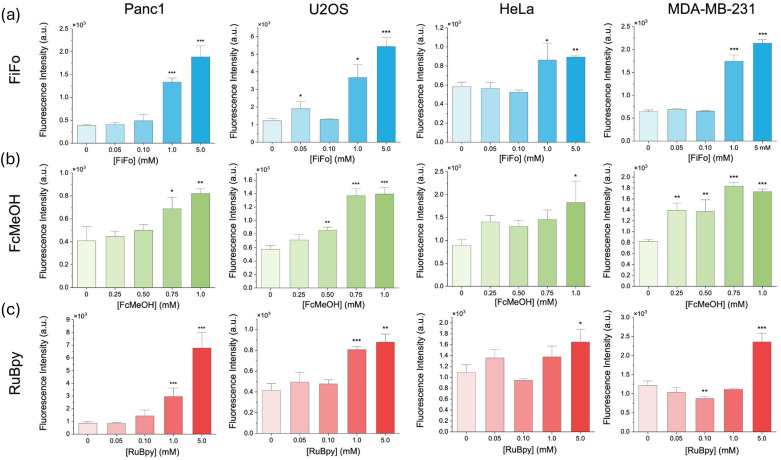
Reactive oxygen species in different cell cultures after incubation in different electrochemical mediators for 6 hours. Fluorescence intensity of ROS stain in different cells lines in (a) FiFo, (b) FcMeOH, and (c) RuBpy. Mean signal intensity of the ROS fluorescent dye (ex/em 485 nm/520 nm) was detected using flow cytometry and is provided as a function of mediator concentration (*n* = 6). All treatments were compared to the control (0 mM) *p < 0.05. **p < 0.01. ***p < 0.001.

In this study, we set out to see if redox mediators upregulate the production of intracellular reactive oxygen species in key cell lines. To quantify the relationship between mediator concentration and reactive oxygen species, we used flow cytometry for a high throughput analysis of intracellular ROS levels in cell populations exposed to redox mediators. To mitigate the effect of confounding variables on ROS generation, cell culture conditions were carefully controlled and kept constant throughout all the experiments. The cells were seeded in 6-well cell culture plates at the same density and volume within each experiment and grown at a constant temperature and carbon dioxide partial pressure of 37 °C and 5%, respectively. This allowed us to compare the baseline ROS levels in control (0 mM) samples with samples containing different concentrations of mediators. Briefly, each cell line was grown in flask till 80%–90% confluence and then moved to six well plates where they were left to adhere overnight. The cells were then incubated for six hours in mediator after which they were treated with CellROX dye for ROS-correlated fluorescence. Upon imaging, the cells were lifted with trypsin and quantified using flow cytometry. The cell-permeable CellROX dye is weakly fluorescent while in a reduced state but emits a bright green photostable fluorescence upon oxidation by ROS and subsequent binding to DNA.^57^ The ROS observed after each treatment was compared to the control (0 mM) treatment. We found that all three mediators, FiFo, FcMeOH, and RuBpy, resulted in a substantial increase in ROS across all cell lines when exposed to the highest concentration of mediator (Fig. 1), which was 5 mM for FiFo and RuBpy, and 1 mM for FcMeOH. Mediator-induced ROS production has previously only been shown for HepG2 cells in 0.60 mM FcMeOH.^25^ Our work extends this observation over several concentrations and cell types. FiFo and FcMeOH also caused a significantly higher amount of reactive oxygen species for the next highest concentrations, 1 mM FiFo (Fig. 1a) and 0.75 mM FcMeOH (Fig. 1b), for all cell lines, except HeLa in FcMeOH. 1 mM RuBpy only caused a significant increase in ROS in Panc1 and U2OS cells (Fig. 1c). The highest increase in ROS was seen in U2OS cells exposed to 5 mM FiFo and Panc1 cells exposed to 5 mM RuBpy. HeLa cells in general appear to be less sensitive to the redox mediators as only the highest concentrations of mediator, except for FiFo, caused an increase in ROS. This agrees with previous studies that show HeLa cells are more resistant to external perturbations than other cell lines.^58^ Overall, all cell lines showed a significant increase in reactive oxygen species with increasing concentration of the redox mediators.

### Cell migration

Cell migration is a key process in the livelihood and development of multicellular organisms. Essential physiological processes depend on cell motility including embryonic development, tissue repair and regeneration, and disease progression.^59^ Cells change their movement based on their phenotype as well as the surrounding microenvironment.^60^ Thus, cell motility is a rather complex phenomenon affected by many internal and external factors. Underlying mechanisms, including the regulation of cell adhesion and cytoskeletal dynamics, are different between normal and malignant cells.^61^ In cancer cells, it is the plasticity of these mechanisms that perpetuates migration and dissemination in stressful microenvironmental conditions.^61^ For example, carcinoma cells often undergo epithelial to mesenchymal transition, increasing their ability to migrate and invade surrounding tissues.^62^

**Fig. 2:**
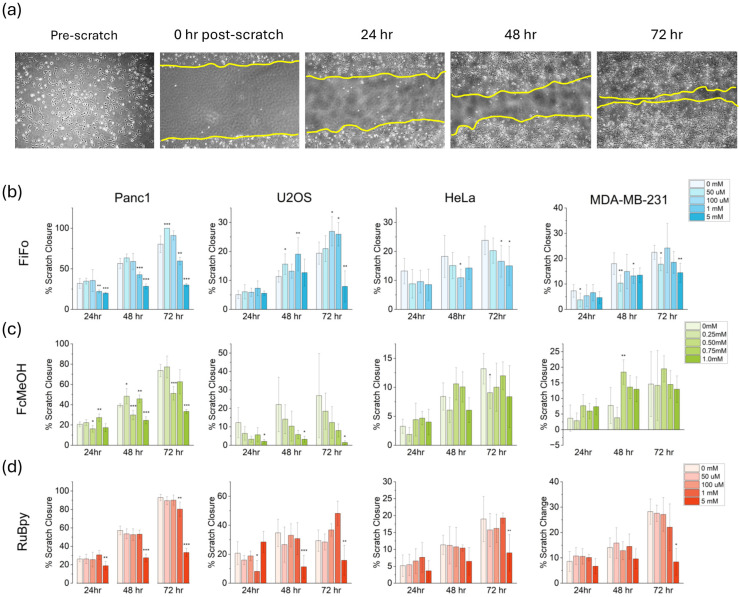
Cell migration in different cell cultures during incubation in different electrochemical mediators over a 72 hours period. Images showing the migration of Panc1 cells over a 72 hours period with the outline of the cellular front in yellow (a). FiFo (b), FcMeOH (c), and RuBpy (d) were studied in Panc1, U2OS, HeLa, and MDA-MB-231 cells. Percent closure of the scratch was quantified using ImageJ (*n* = 6). All treatments were compared to the control (0 mM) *p < 0.05. **p < 0.01. ***p < 0.001.

Herein, we tracked cell migration in the presence of redox mediators over the course of 72 hours, to gain further insight into the impact of redox mediators on the mobility of different cell lines. Briefly, cells were grown in six well plates till the desired confluence was achieved after which a scratch was made across the center of the plate and brightfield images were taken to record scratch margins. The cells were then incubated in the presence of different mediators and scratch closure was observed over time (Fig. 2a). The cell migration in each mediator treatment was compared to the control (0 mM) treatment. Unlike the ROS studies, trends in cell migration were significantly different across specific cell types or mediator species, with the exception of Panc1. This is likely because absolute cell migration (percent scratch closure) was by far the highest in Panc1 cells (Fig. 2), so migration dependence on mediator concentration was more pronounced. Motility was hindered for Panc1 in 1 mM and 5 mM FiFo, 0.75 mM and 1 mM FcMeOH, and 5 mM RuBpy at nearly all timepoints. In contrast, HeLa cells showed the least absolute cell migration, followed closely by MDA-MB-231. These lesser motile cell lines did not show significant changes in migration until 72 hours in 5 mM FiFo or 5 mM RuBpy. Lastly, U2OS cells showed a clear decrease in cell migration in 1 mM FcMeOH and 5 mM RuBpy at nearly all timepoints. FiFo only decreased migration of U2OS in 5 mM FiFo after 72 hours. All cell lines used in this study are immortalized, meaning they likely possess genomic instability and high mutation rates. Increasing evidence shows that genetic mutations might accelerate migration rates of cancer cells,^63^ which may explain the striking differences in migration rate between the different cell lines we used.

With respect to the specific mediator species, FcMeOH resulted in the least cell migration difference overall (Fig. 2b). This may be due to the ability of FcMeOH to pass through the cell membrane and directly affect intracellular mechanics. Of the three mediators used in this work, FcMeOH is the only compound partially permeable to the cell membrane,^64^ due to its relatively hydrophobic nature. Ferrocyanide, ferricyanide, and tris(bipyridine) ruthenium(ii) chloride are not able to pass through the cell membrane due to their charge state and hydrophilicity. In all cell lines, 5 mM concentrations of both RuBpy and FiFo showed compromised cell migration by the 72-hour timepoint. However, this may be reflected by increased cell death rather than decreased cell migration, since exposure to 5 mM concentrations for 72 hours showed significant cell mortality (Fig. S3†). Overall, cell migration was compromised at the highest concentrations of mediator (5 mM FiFo, 1 mM FcMeOH, and 5 mM Rubpy) in all cell lines. This suggests that concentrations of FiFo and RuBpy up to 1 mM and FcMeOH up to 0.75 mM for as long as 72 hours do not have a significant effect on cell migration. Some cells such as U2OS in FcMeOH (Fig. 2c), show high variability in percent scratch closure. This extreme variability likely arises from the inherent differences associated with cells moving into a relatively large empty area, which is a rather random process. However, with *n* = 6 replicates, we are still able to observe the cell migration trends over time.

### Cell growth and recovery

**Fig. 3:**
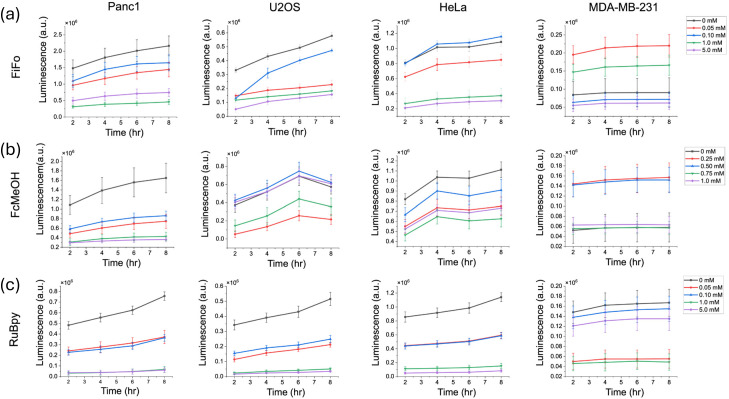
Cell growth in different cell cultures during incubation in different electrochemical mediators over an 8 hours period. FiFo (a), FcMeOH (b), and RuBpy (c) were studied in Panc1, U2OS, HeLa, and MDA-MB-231 cells. Cell growth was quantified by measuring the luminescence produced from luciferase enzyme over time (*n* = 6). The control luminescence signal was subtracted from each experimental sample to correct for background luminescence.

Cells from multicellular organisms rely on signals from exogeneous compounds for survival and growth. Cell livelihood is controlled by the inhibition of apoptosis or promotion of cell survival.^65^ Cell growth assays are an essential part of cell viability analyses as they provide a measure of how a compound affects the ability of cells to proliferate and maintain a population of robust cells. It is important to take into consideration that cancer cells exhibit a unique ability to evade growth suppressors and apoptotic signaling, which increases their overall growth rates.^66^ Therefore, we would expect non-cancerous cell growth rates to be particularly vulnerable to redox mediator induced changes as they possess a lower growth and mutation rate. To investigate our hypothesis, luminescence was measured over time in a noncancerous cell line, HEK 293. As seen through our study on the four cancerous cell lines in Fig. 3, luminescence increased gradually over an 8 hours period, indicating cell proliferation and an increase in the number of viable cells. In contrast, luminescence in HEK 293 was overall lower than in cancerous cells and decreased dramatically for both the control (0 mM) and mediator exposed samples after reaching a maximum luminescence around 6 hours (Fig. S5†). This may be due to the HEK 293 reaching a maximum confluence around 6 hours at which point the culture entered the decline phase of cell growth, leading to a lowered number of viable cells in all the samples, regardless of exposure to mediator.

In this study, we investigated the impact of mediator compounds on cell growth by using a luciferase enzyme-based cell viability assay to monitor cell viability in the same sample over time. This assay relies on a cell permeant substrate that is reduced intracellularly. Upon activation inside the cells, the substrate reacts rapidly with luciferase. Since only viable cells can reduce the substrate, the luminescence signal is related to the number of viable cells and the cellular reducing potential. An increase in luminescence can reasonably be correlated to cell growth as there must be an increase in the total number of viable cells for luminescence to increase. However, a decrease or zero change in luminescence can be correlated to a change in cell proliferation and/or a change in cell death. Based on our ROS results, it is more likely that a decrease in luminescence at the highest concentrations of mediator is due to ROS-induced cell death. To obtain these results, cells were seeded in 96-well plates at approximately 80% confluence. A solution of mediator dissolved in cell media containing the luciferase enzyme and substrate were then added to the cells. Luminescence was measured every 2 hours over an 8-hour incubation period. As a control, background luminescence signal was measured in untreated cells without the luminescent-producing enzyme and substrate. In Fig. 3, the reported luminescence values are the difference between the luminescence of each treatment and the background signal.

For nearly every cell line and mediator, cell growth was substantially lower at the highest concentrations of each mediator, indicating that the presence of a redox mediator dramatically lowers the total number of viable cells (Fig. 3). In general, as concentration of FiFo and RuBpy increased, a decrease in cell viability was observed in all cell lines, except MDA-MB-23 (Fig. 3a and c). In particular, the 1 mM and 5 mM concentrations of FiFo and RuBpy caused a substantial decrease in luminescence, except in MDA-MB-231. RuBpy displayed the most striking disparities in the dependence of cell viability on mediator concentration, as the presence of RuBpy led to significantly less growth in all samples apart from the control (Fig. 3c) in all cell lines except MDA-MB-231. Additionally, 1 mM FcMeOH caused a substantial decrease in growth for all cell lines except HeLa, which did not show a concentration-dependent response of cell growth (Fig. 3b). FcMeOH showed the most variable growth response with respect to concentration. As implied previously in our cell migration assay, this may be due to the ability of FcMeOH to pass through the cell membrane. This phenomenon may cause variable concentrations of FcMeOH inside and outside the cell and directly interfere with intracellular regulation of cell reduction potential. However, the luciferin substrate is dosed in high amounts for live cell experiments to overcome any permeability issues.^67^ In addition, there is no evidence to suggest that FcMeOH could be an inhibitor of the luciferase–luciferin reaction as the structure of FcMeOH does not resemble any known inhibitors of luciferase.^68^ Overall, our results do not show specifically whether a decrease in the total number of viable cells is due to a decrease in cell growth or an increase in cell death.

It is important to note that MDA-MB-231 and U2OS cells produced a much lower luminescence signal overall compared to the other cell lines used. MDA-MB-231 signals were an order of magnitude lower than signals in Panc1 and HeLa while the U2OS signals were about four times lower than both Panc1 and HeLa signals. This may be due to the relatively smaller cell size of 15 μm or less in diameter for U2OS and MDA-MB-231 as compared to Panc1 and HeLa which are roughly 20–40 μm in diameter.^69,70^ This suggests that U2OS and MDA-MB-231 may not be able to turn over the substrate as rapidly as Panc1 and HeLa, leading to a lower enzymatic activity and luminescent signal. Furthermore, the lower luminescence signal may be the reason that a clear concentration gradient trend was not observed for MDA-MB-231 cells.

In addition to monitoring cell viability over time and directly in the presence of redox mediator, we monitored the recovery of cells in fresh cell media after exposure to redox mediator in a separate set of experiments done on separate samples. First, cells were exposed to the mediator for a period of 6 hours. The mediator was then removed and diluted with blank media three times over. The enzyme and substrate were added to the cells in the final addition of media and the luminescence was tracked over a 24-hour period, with luminescence measurements taken 6 hours, 18 hours and 24 hours following the addition of blank media.

**Fig. 4:**
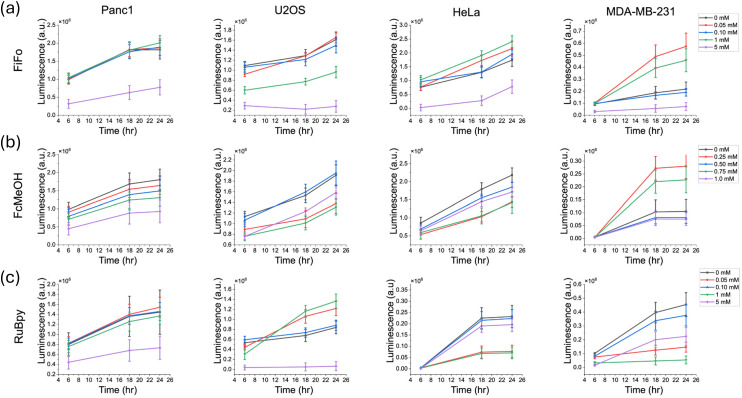
Cell recovery in different cell cultures after incubation in different electrochemical mediators for a 6-hour period. Recovery was measured over a period of 24 hours with measurements taken at 6 hours, 18 hours, 24 hours. FiFo (a), FcMeOH (b), and RuBpy (c) were studied in Panc1, U2OS, HeLa, and MDA-MB-231 cells. Cell growth was quantified by measuring the luminescence produced from luciferase enzyme activity over time (*n* = 6). The control luminescence signal was subtracted from each experimental sample to correct for background luminescence.

For FiFo, cell viability remained significantly lower in 5 mM FiFo for all cell lines (Fig. 4a). For FcMeOH, the recovery of cell viability showed no clear trend in any of the cell lines, except for Panc1, which showed a clear decrease in luminescence across concentration (Fig. 4b). For RuBpy, Panc1 and U2OS showed significantly lowered cell viability across time in the 5 mM concentration (Fig. 4c). In contrast, HeLa and MDA-MB-231 did not show clear trends with respect to cell viability *versus* RuBpy concentration. For FcMeOH, with the exception of Panc1, which showed a clear decrease in recovery across concentration, recovery was variable across U2OS, HeLa, and MDA-MB-231 as was also observed in the growth assays.

For some of the assays in this cell recovery analysis, the luminescence seemed to vary with respect to mediator concentration. This may be due to greater natural variation in cell growth rates between samples, since the starting confluence was only about 50%. Since samples at a lower confluence have a greater capacity to grow, even small differences in starting confluence could have a greater impact on the subsequent growth profile, especially in a 96-well plate. For instance, for HeLa and MDA-MB-231 the luminescence after 6 hours of recovery was largely the same at each concentration of mediator. At 18 hours and 24 hours of recovery however, luminescence varied across concentration.

The concentrations of mediator that most substantially alter different cellular behaviors are shown in Table 1. Scientists looking to use the reported mediators in their studies should aim to optimize the concentration of mediators to remain below the value presented. In addition to concentration of mediator, it is crucial to consider the time of exposure to mediator. In this study, reactive oxygen species and cell viability were monitored in the range of 6–8 hours. Cell migration was monitored over 72 hours. In the migration study, although cells continued to fill the scratch over time, substantial cell death was observed after 72 hours of exposure to high concentrations of mediator (Fig. S3†). Lower concentrations in the micromolar range appeared to not cause substantial cell death even after 72 hours. We hypothesize that exposure times much shorter than those in the scope of this study (*e.g.* 30 minutes) would not detrimentally affect cell health at the reported concentrations.

**Table 1:** Guideline for mediator concentrations to avoid in electrochemical studies on living cells. The values shown are the concentrations at which different measures of cell health were most significantly impacted in four different cell lines. The value or range of values reported for cell ROS and migration are statistically significant at *p* < 0.05. The value reported for cell viability is the concentration associated with the lowest observed luminescence value over time

Redox mediator	Cell line	Cell ROS	Cell migration	Cell viability
FiFo	Panc1	1 mM	5 mM	1 mM
	U2OS	1 mM	5 mM	5 mM
	HeLa	1 mM	5 mM	5 mM
	MDA-MB-231	1 mM	5 mM	5 mM
FcMeOH	Panc1	0.75 mM	1 mM	1 mM
	U2OS	0.50 mM	1 mM	0.25 mM
	HeLa	1 mM	N/A	0.75 mM
	MDA-MB-231	0.25 mM	N/A	0.75 mM
RuBpy	Panc1	1 mM	5 mM	1 mM
	U2OS	1 mM	5 mM	1 mM
	HeLa	5 mM	5 mM	1 mM
	MDA-MB-231	5 mM	5 mM	1 mM

## Conclusion

In this work, we carry out the first comprehensive study on the effects of redox mediators on cell health. We investigated cell health on four commonly used cell lines in the presence of three different redox mediators. Cell health was examined through three different lenses: oxidative stress, cell migration, and cell growth. As concentration of mediator increased, reactive oxygen species increased across all cell lines and redox mediators. In the growth assays, cell growth decreased across mediator concentration in general, especially at the highest concentration of mediator. Lastly, cell migration was only hindered at the highest concentration of mediator for nearly every cell line and mediator. Together, these indicators of cell viability paint a broad picture of the health of cells exposed to redox mediators with respect to both concentration and exposure time.

Most importantly, this study provides a benchmark for redox mediator concentrations that can be used in biological studies without significantly impacting cell viability. Based on our results, we recommend using ferrocyanide/ferricyanide concentrations of no more than 100 μM. Ferrocene methanol concentrations should not exceed 500 μM. Tris(bipyridine) ruthenium(ii) chloride concentrations should not exceed 100 μM concentrations. For applications that require many hours of mediator exposure to cells, such as electrochemical-based sensors that can be deployed as medical devices, concentrations no greater than 100 μM are recommended to mitigate perturbations to the redox state of the cell. If redox mediator exposure periods of only a few hours or less are used, commonly associated with SECM and ECL, then higher concentrations of mediator such as 1 mM can potentially be used. Additionally, this study clearly shows that 5 mM concentrations of FiFo and RuBpy significantly impact cell health. We hope this work will serve as an experimental guideline for bioanalytical studies using redox mediators in the presence of living cells.

## Author contributions

Samuel P. Nortz—formal analysis, investigation, visualization, writing (original draft), and writing (review and editing). Vanshika Gupta—conceptualization, methodology, writing (review and editing). Jeffrey E. Dick—conceptualization, supervision, and writing (review and editing).

## Data availability

Data for this article including luminescence data (.xlsx), scratch assay data (.xlxs), and flow cytometry data (.fcs files) are available at the Open Science Framework data repository titled “Impact of Common Redox Mediators on Cellular Health: A Comprehensive Study” at https://osf.io/tr3x9/.

## Conflicts of interest

The authors declare no conflicts of interest.

## Supplementary Material

AN-150-D5AN00017C-s001
