# Supplementary material for: The impact of common redox mediators on cellular health: a comprehensive study
Source: Analyst. 2025 Mar 12;150(9):1795–806. doi: 10.1039/d5an00017c (PMC11966090; doi:10.1039/d5an00017c)
Supplement: AN-150-D5AN00017C-s001 [file AN-150-D5AN00017C-s001.pdf]

Supplementary Information (SI) for Analyst.
This journal is © The Royal Society of Chemistry 2025

Electronic Supporting Information

Impact of Common Redox Mediators on Cellular Health: A Comprehensive Study

Samuel Nortz[a], Vanshika Gupta[a], Jeffrey E. Dick[a,b*]

[a]Department of Chemistry, Purdue University, West Lafayette, IN 47907, USA
[b]Elmore Family School of Electrical and Computer Engineering, Purdue University, West Lafayette, IN 47906, USA
***To whom correspondence should be addressed: jdick@purdue.edu**

Table of Contents

S1. Flow Cytometry Dot Plots…………………………………………………………..…....2

S2. Flow Cytometry Raw Data………………………………………………………….....3

S3. HeLa Cell Images……………………………………………………………………4

S4. pH Measurements of Mediators……………………………………………………..5-6

S5. Luminescence of HEK 293 cells in Mediators……………………………………...…7-8

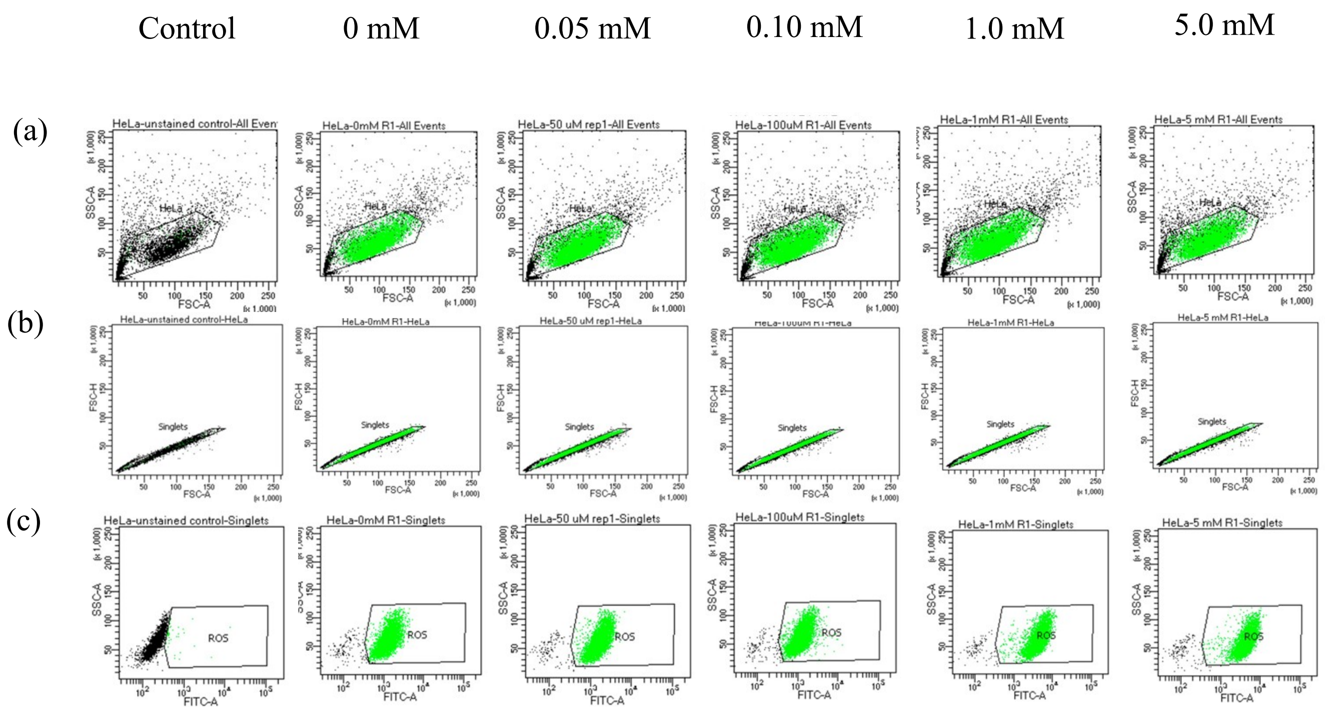

Figure S1. Example of two-dimensional scatter plots of flow cytometry data of HeLa cells in FcMeOH. (a) Forward scattering (FSC) which shows the relative size of a cell versus side scattering (SSC) which shows the relative complexity of a cell. (b) FCS area versus FCS height to negate doublets. (c) FITC fluorescence intensity versus SSC area.

Sample	Population	# Events	% Parent	FITC-A Mean
Control	All events	10000	-	-
	HeLa	6651	66.5	314
	Singlets	2906	43.7	564
	ROS	44	1.5	1239
	**Population**	**# Events**	**% Parent**	**FITC-A Mean**
0 mM	All events	10000	-	-
	HeLa	7800	78	1171
	Singlets	6098	78.2	1267
	ROS	5945	97.5	1296
	**Population**	**# Events**	**% Parent**	**FITC-A Mean**
0.05 mM	All events	10000	-	-
	HeLa	7853	78.5	1487
	Singlets	5996	76.4	607
	ROS	5864	97.8	1640
	**Population**	**# Events**	**% Parent**	**FITC-A Mean**
0.10 mM	All events	10000	-	-
	HeLa	7917	79.2	1131
	Singlets	6131	77.4	1224
	ROS	5990	97.7	1249
	**Population**	**# Events**	**% Parent**	**FITC-A Mean**
1.0 mM	All events	10000	-	-
	HeLa	7643	76.4	3777
	Singlets	5660	74.1	4330
	ROS	5556	98.2	4408
	**Population**	**# Events**	**% Parent**	**FITC-A Mean**
5.0 mM	All events	10000	-	-
	HeLa	7445	74.5	3490
	Singlets	4917	66	4363
	ROS	4770	97	4492

Figure S2. Output of flow cytometry quantitative data of HeLa in FcMeOH. Population distribution of all events, HeLa, singlets, and ROS are provided based on the selected gating in Figure S1. The average of the triplicates of FITC-A of ROS is reported in Figure 1.

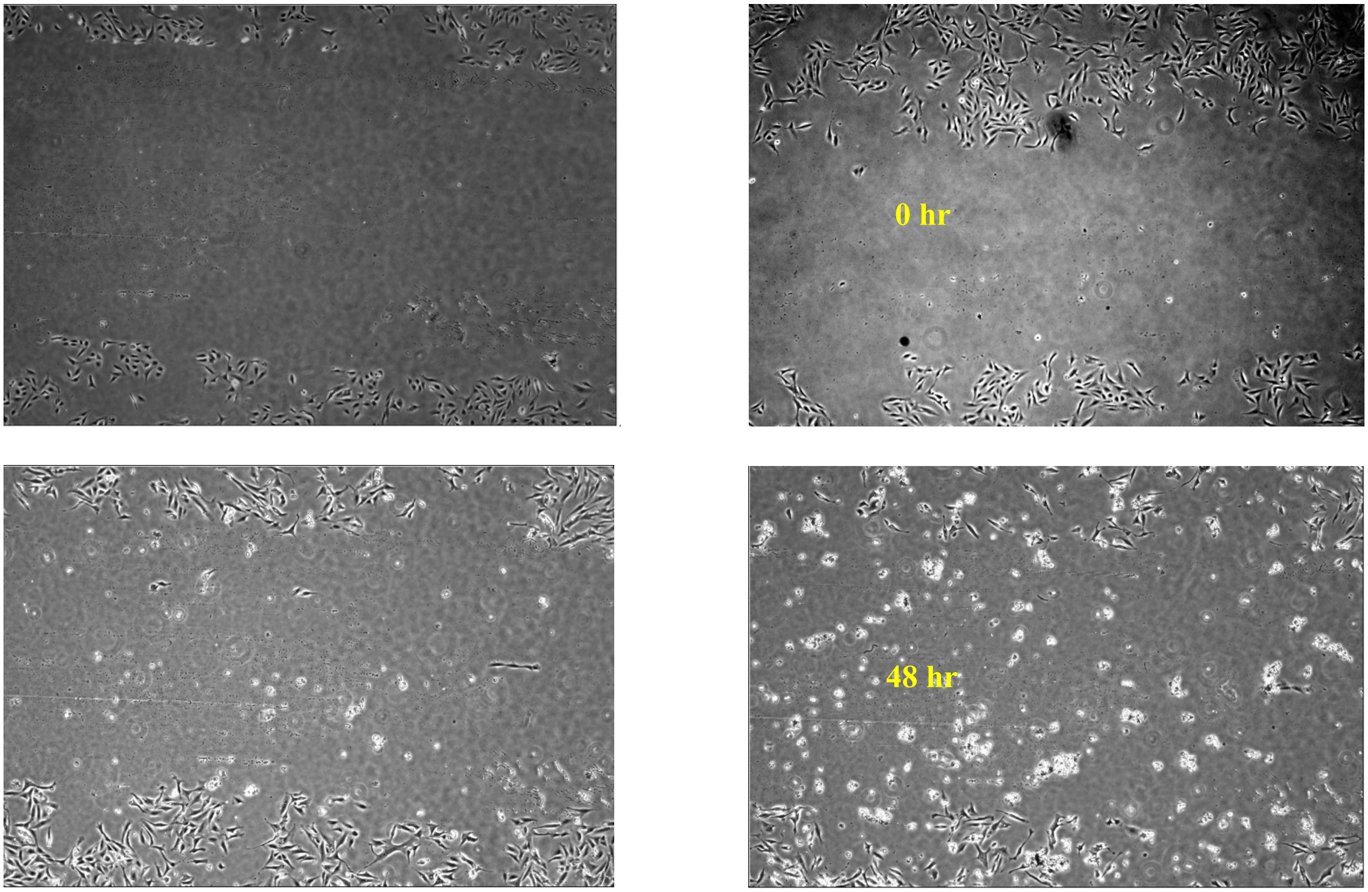

Figure S3. Migration of U2OS cells in 5 mM FiFo. The size of the scratch only appears to decrease over the first 24 hours. After 48 hours of exposure to the mediator, the cells begin to die and the cellular front diminshes. When the cell's die, they detach from the bottom of the plate and shrivel up, appearing as white dots in the above images.

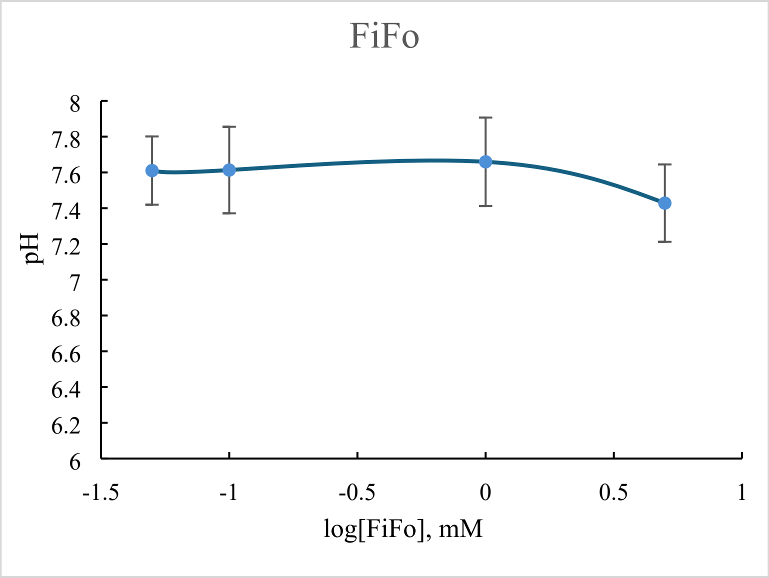
FiFo
8
7.8
7.6
7.4
7.2
7
6.8
6.6
6.4
6.2
6
pH
-1.5
-1
-0.5
0
0.5
1
log[FiFo], mM

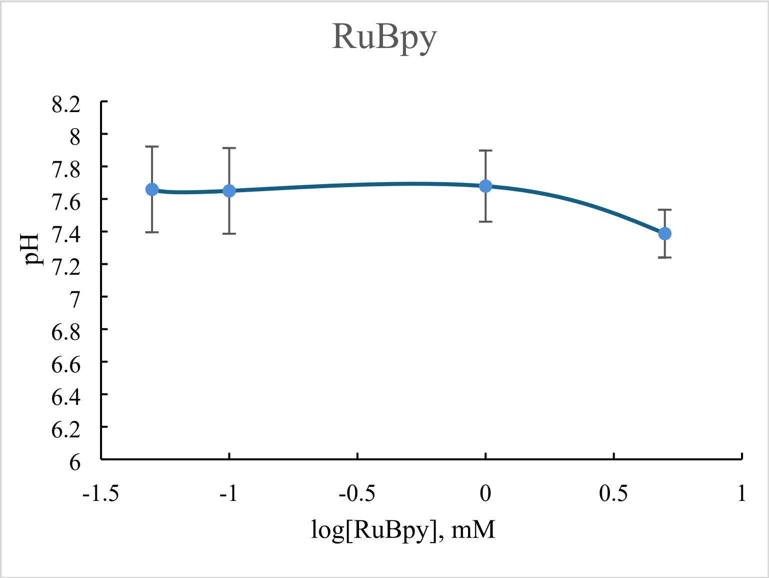
RuBpy
8.2
8
7.8
7.6
7.4
7.2
7
6.8
6.6
6.4
6.2
6
pH
-1.5
-1
-0.5
0
0.5
1
log[RuBpy], mM

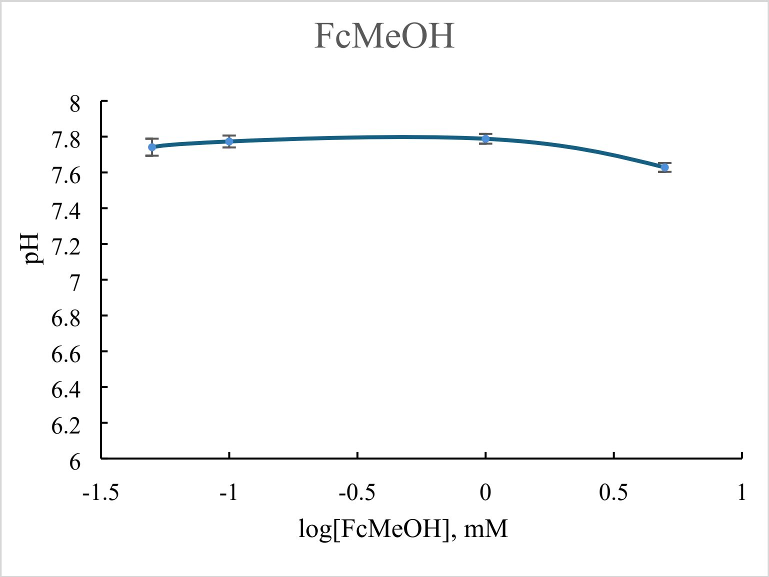

Figure S4. pH measurements (n=3) of all concentrations of mediator solutions used in this work. The pH of the blank DMEM cell media solution is 7.35. Average pH values for ferro/ferricyanide and tris(bipyridine) ruthenium (II) chloride were around 7.6. Ferrocene methanol varied the greatest in pH with all concentrations being slightly basic and 0.75 mM deviating from the baseline pH the most (pH=7.82).

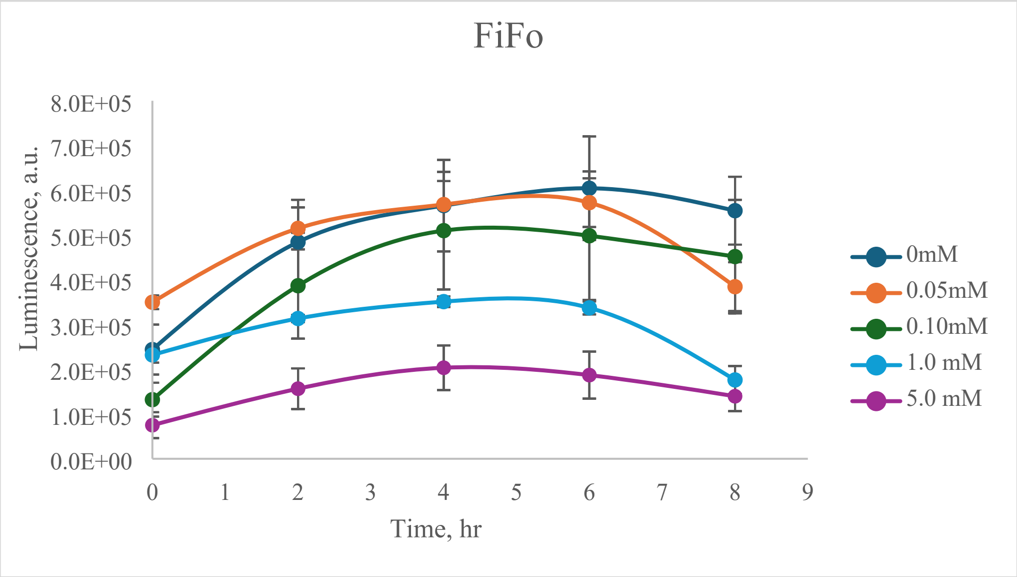
FiFo
8.0E+05
7.0E+05
6.0E+05
5.0E+05
4.0E+05
3.0E+05
2.0E+05
1.0E+05
0.0E+00
Luminescence, a.u.
0
1
2
3
4
5
6
7
8
9
Time, hr
0mM
0.05mM
0.10mM
1.0 mM
5.0 mM

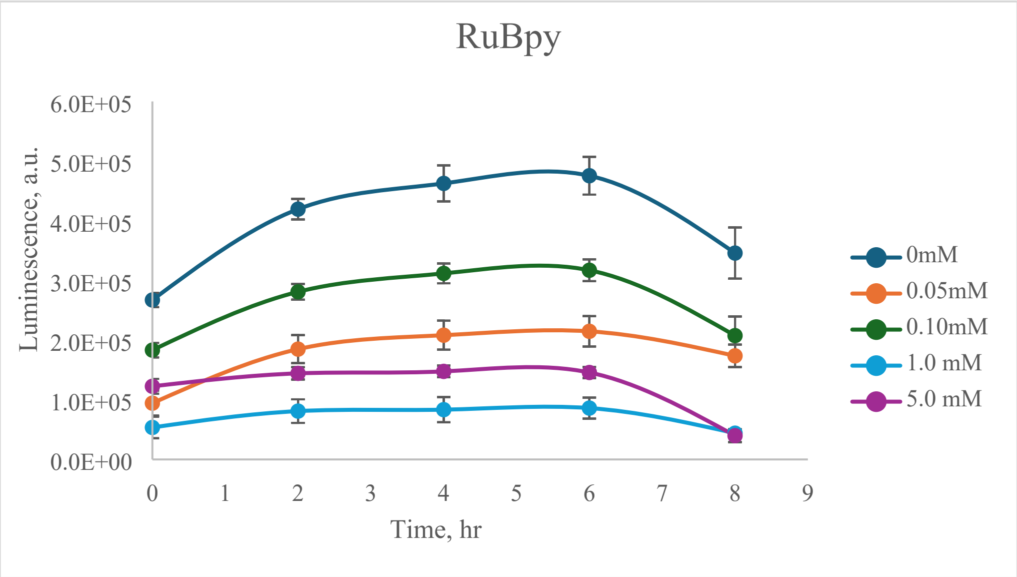
RuBpy
6.0E+05
5.0E+05
4.0E+05
3.0E+05
2.0E+05
1.0E+05
0.0E+00
Luminescence, a.u.
0
1
2
3
4
5
6
7
8
9
Time, hr
0mM
0.05mM
0.10mM
1.0 mM
5.0 mM

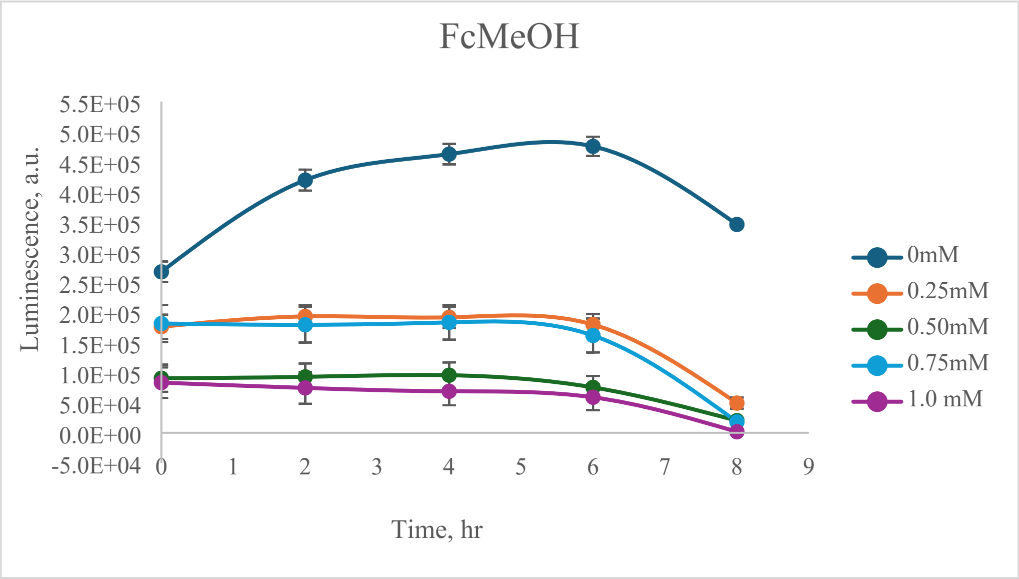

Figure S5. Luminescence measurements in HEK 293 cells exposed to FiFo, RuBpy, and FcMeOH over 8 hours. Luminescence was measured for n=6 replicates at each concentration of mediator.
